# Alterations of the neural substrate in childhood apraxia of speech: new evidence from neuroimaging

**DOI:** 10.1093/braincomms/fcaf302

**Published:** 2025-09-16

**Authors:** Paolo Bosco, Irina Podda, Emilio Cipriano, Clara Bombonato, Paola Cipriani, Mariaelisa Bartoli, Rosa Pasquariello, Simona Fiori, Laura Biagi, Anna Chilosi

**Affiliations:** IRCCS Stella Maris Foundation, Pisa 56128, Italy; Parole al Centro Studio di Logopedia, Genova 16129, Italy; IRCCS Stella Maris Foundation, Pisa 56128, Italy; Department of Neuroscience, Rehabilitation, Ophthalmology, Genetics, Maternal and Child Health (DINOGMI), University of Genoa, Genoa 16132, Italy; IRCCS Stella Maris Foundation, Pisa 56128, Italy; IRCCS Stella Maris Foundation, Pisa 56128, Italy; Unità Funzionale Salute Mentale Infanzia e Adolescenza (UFSMIA), Azienda Usl Toscana Nord Ovest, Pisa 56124, Italy; IRCCS Stella Maris Foundation, Pisa 56128, Italy; Department of Neuroscience, Meyer Children’s Hospital, Florence 50139, Italy; IRCCS Stella Maris Foundation, Pisa 56128, Italy; IRCCS Stella Maris Foundation, Pisa 56128, Italy

**Keywords:** neurodevelopment, somatosensory cortex, morphometric brain measures, white matter diffusion, neuroimaging

## Abstract

Childhood apraxia of speech is a motor speech disorder characterized by deficits in programming, planning, and movement control, necessary for speech production with severe impact on oral and written language. Currently there are few studies on how speech is functionally rooted in neuroanatomy in children with apraxia of speech. The present cross-sectional study aimed at further identifying the specific neuroanatomical substrate of childhood apraxia of speech and at analysing the relationship between Magnetic Resonance Imaging findings and speech measures in a relatively large group of Italian children with this disorder. The brain structures of 71 children with apraxia of speech were compared to those of 30 age-matched typically developing peers. For each subject, the morphological brain images were segmented according to a specific atlas, and cortical thickness and volume measures were extracted from cortical and subcortical structures, respectively. Moreover, using voxel-based morphometry with a diffeomorphic anatomical registration procedure, grey matter morphometry of the two groups was compared. We also used diffusion weighted imaging (in 67 out of 71 children with apraxia of speech and all typically developing peers) to investigate the white matter integrity in specific speech-language-related tracts, comparing the mean fractional anisotropy along the tracts. Children with apraxia showed significantly greater grey matter volumes and increased cortical thickness than their typically developing peers in several areas involved in speech and language processing. After correction for multiple comparisons, significant increments in cortical thickness and volume survived in the left postcentral gyrus and bilaterally in the thalami. The diffusion study revealed a significant reduction of fractional anisotropy in childhood apraxia of speech with respect to typically developing children in the left frontal aslant tract in both supplementary motor area and pre-supplementary motor components. Speech severity and diadochokinetic rate of children with apraxia of speech correlated significantly with cortical thickness and volume measures extracted from the rostral middle-frontal gyrus, the left precuneus, and the left thalamus. The same speech measures correlated also with the fractional anisotropy values along the frontal aslant tract. Our results revealed grey matter alterations in childhood apraxia of speech, in a widespread circuit of cortical and subcortical areas, and in particular in both thalami and the left postcentral gyrus, crucial regions for sensorimotor control. Moreover, diffusion study results also provided further support to the involvement, in childhood apraxia of speech, of the left frontal aslant tract, a fundamental pathway for speech movement planning and programming.

## Teaser

Bosco *et al*. have studied children with apraxia of speech, finding cerebral structural differences in key regions for speech/language and motor tasks (thalamus, postcentral gyrus). They also found reduced white matter integrity in a tract vital for speech planning. These brain changes correlate with speech severity.

## Graphical Abstract

**Graphical Abstract:**
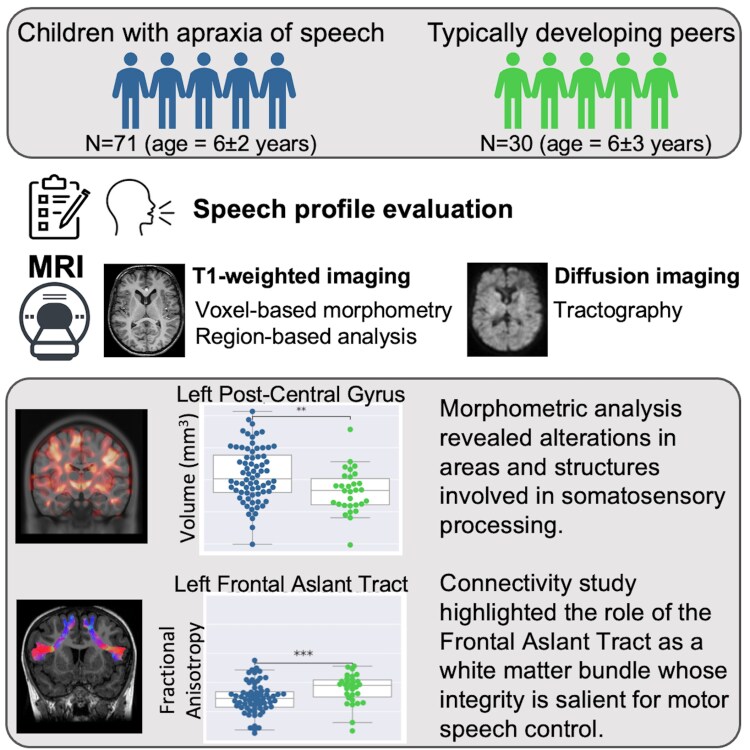

## Introduction

Childhood Apraxia of Speech (CAS) is a motor speech disorder characterized by deficits in programming, planning, and movement control, necessary for speech production^1–3^ in children without neuromuscular deficits.^4^ Motor programming/planning is the process of transformation of an abstract phonological code into kinematic commands for speech. Auditory and somatosensory feedback play a crucial role, both during acquisition and in adult production.^5–9^

A body of neuroimaging studies has investigated how speech and language are functionally rooted in neuroanatomy, but only a few of them have considered children with CAS. Different from acquired apraxia of speech in adults, the majority of children with CAS do not show significant structural brain anomalies at conventional Magnetic Resonance Imaging (MRI),^10–12^ though there is some evidence of brain abnormalities at a microscopic level. Moreover, possibly due to small samples, the results are rather inconsistent across the studies, in particular with regard to brain-function correlations.

Kadis *et al*.^13^ measured the cortical thickness in 14 children with CAS and 14 typically developing (TD) peers. They found that children with CAS presented thicker cortex in the left posterior supramarginal gyrus, a parietal area known to be involved in adults in phonological processing of spoken and written language. The authors could not find any correlation with speech measures, but interestingly, they found that after receiving speech motor therapy that intensively employs auditory, visual and, especially, somatosensory cues, children with CAS showed a reduction of cortical thickness in the left superior temporal gyrus. Increased cortical volumes were detected by Conti *et al*.^14^ in the supramarginal gyrus, as well as in the frontal lobe (paracentral lobule and pars triangularis of the inferior frontal gyrus) and in the putamen, in children with CAS compared to TD, but the authors did not investigate possible correlations with speech performances. Preston *et al*.^15^ found that children with residual Speech Sound Disorders (SSD) (but no CAS) showed greater grey matter volumes in the superior temporal gyrus bilaterally relative to TD controls. In these studies, the volumetric alterations, either in children with CAS or with SSD, were interpreted as the result of inadequate synaptic pruning during the maturation of the neurodevelopmental substrate.

Studies on structural brain connectivity provide further evidence on brain organization in TD children and with CAS. According to the dual stream model,^16–20^ the dorsal temporo-parietal-frontal stream, which comprises the arcuate fasciculus, is suggested to process auditory speech-to-articulation transformations, including feedback necessary to predict the speech output, as well as online error detection and correction.^20,21^ Dorsal stream alterations were actually found by Liégeois *et al*.^22^ in a study on a family aggregation of CAS without language impairment, in which 7 affected children presented with white matter reduction in the arcuate fasciculus bilaterally. Fiori *et al*.^23^ using Diffusion Weighted Imaging (DWI) for a connectomic approach to the study of microscopic brain properties in CAS, found reduced white matter connectivity compared to TD controls in three subnetworks, which included connections between cortices, whose role is ascertained in speech and language processing, as well as in auditory and somatosensory processing. More recently, Bombonato *et al*.^24^ reported decreased Fractional Anisotropy (FA) in the pre-Supplementary Motor Area (SMA) component of the Frontal Aslant Tract (FAT) in a group of 30 preschool children with CAS compared to 30 TD peers, and found a correlation between severity of speech and decreased connectivity in both FAT components. The FAT is a frontal white matter tract that connects the SMA and the pre-SMA to the inferior frontal gyrus, namely with the opercular and triangular parts, and the anterior insula.^25,26^ The left FAT is involved in planning and programming of sequential movements, including speech, in speech initiation and verbal fluency,^5,27^ whilst the right FAT is more involved in general motor control, regulating competition between sequential motor plans and contributing to the suppression of inappropriate automatic responses.^27^ Moreover, the FAT also appears to play a key role in executive functions, particularly in inhibitory control and action conflict monitoring. This dual hemispheric specialization of the FAT could explain both the linguistic and executive difficulties observed in children with CAS.^12,24^

The present study aimed at further identifying the specific neuroanatomical substrate of CAS and at analysing the relationships between MRI findings and speech measures in a relatively large group of Italian children with CAS. We used brain morphometry to compare, both at the Region of Interest (ROI) and voxel level, the cerebral grey matter volumes of a notable number of children with CAS to those of a group of age-matched typically developing children. We also used DWI to investigate the white matter integrity in specific speech and language-related tracts, comparing the mean FA values along these tracts in the two groups.

We hypothesize that children with CAS may present significant morphometric differences compared to typically developing peers in brain areas known to be crucial for speech and whose alteration has already been reported in CAS. These areas include the left cortical motor system, as well as sensory processing cortices and subcortical structures. With regard to the connectivity study, we hypothesize the involvement in CAS of white matter bundles that connect speech and language areas, and in particular of the FAT, a tract of increasing interest for its recently suggested role in speech motor disorders.^24^

## Materials and methods

Written parental informed consent and child assent for participation in this study and for data publication were obtained in all cases according to the Declaration of Helsinki. The study was approved by the ethics committee of the IRCCS Fondazione Stella Maris (Number 13/2013) and by the regional paediatric ethics committee (CEP) 19-03-2018/RF2016-02361560. Data collection occurred between 2018 and 2021.

### Participants

Seventy-one Italian children with isolated CAS (57 males, 14 females, mean age = 6.1 ± 2.1 years; range = 2.8–11.2 years) were enrolled in this cross-sectional study. The children underwent a thorough neurological, speech–language, and cognitive assessment. Inclusion criteria were Italian as the primary language, the ability to complete a full neurological assessment as well as speech and language evaluation, and the absence of structural brain alterations at conventional MRI. Exclusion criteria were orofacial structural anomalies potentially affecting speech production, hearing impairment, major neurological impairments, autism spectrum disorder or intellectual disability, and the presence of speech features consistent with dysarthria. CAS was diagnosed based on the presence of the three ASHA consensus criteria^4^ and of at least five out of the ten signs on the Mayo checklist,^2^ detectable across a set of speech measures in three contexts of increasing difficulty, at the syllable, word, phrase and complex discourse level. For further details on the speech and language assessment protocol, see Chilosi *et al*.^12^ and Bombonato *et al*.^24^ Non-verbal IQ was evaluated through the administration of the Wechsler Preschool and Primary Scale of Intelligence, 3rd Edition (WPPSI-III), or the Wechsler Intelligence Scale for Children 4th Edition (WISC-IV), depending on the child’s age. All showed non-verbal IQ within the normal range (PIQ or PRI ≥ 85).

The control group for MRI assessment consisted of 30 age-matched children with typical development and a negative report at conventional MRI examination, carried out for nonspecific reasons, such as headache and visual disturbances (21 males, 9 females, mean = 6.5 ± 2.6 years; range = 2.6–12.7 years). No concerns about speech–language development and academic learning were ever reported for the children included in this group, further defined as TD.

### Speech profile

All the children with CAS underwent a speech examination, which included tasks addressing motor speech performances. The assessment was carried out by Speech–Language Pathologists (SLPs) experienced in the evaluation of speech and language in children with motor speech disorders. The following speech measures were considered: phonetic inventory, phonetic inaccuracy and phonetic inconsistency, syllable omissions and DDK two- and three-syllable rate (DDK2 and DDK3, respectively). Syllable omissions, phonetic inaccuracy and inconsistency were evaluated at the single-word level, using a list of 46 probe words of increasing length and motor complexity. The DDK rate consists of a task of maximum performance rate, in which the child must repeat as fast as possible disyllabic or trisyllabic non-word sequences (i.e./pata/and/pataka/), and it is scored as the number of repeated sequences in 20 s. In order to estimate the overall level of speech proficiency, a composite severity score was calculated based on five speech measures (phonetic inventory, phonetic inaccuracy and inconsistency, syllable omissions, and DDK3). Each measure was assigned a score of 0 when normal or borderline and 1 when deficient. The maximum speech severity score was 5.

### MRI acquisition

MRI data were acquired using a GE 1.5 T Signa (GE Healthcare) scanner at IRCCS Stella Maris Foundation. The MRI acquisition protocol, in addition to the traditional imaging commonly used in clinics, included a 3D 1 mm isotropic T1-weighted structural sequence (Fast Spoiled Gradient Echo, time of repetition (TR)/time of echo (TE) = 12.4/5.2 ms; flip angle (fa) = 13°; field of view (FOV) = 256 mm × 256 mm; matrix = 256 × 256; slice thickness = 1 mm; number of excitations (NEX) = 1) for the study of brain anatomy, and a DWI acquisition, obtained by using a 2D single-shot Spin-Echo Echo Planar Imaging (SE-EPI) sequence (TR/TE = 13000/115.8 ms; FOV = 240 mm × 240 mm; matrix = 80 × 80; slice thickness = 3 mm; NEX = 1) including 30 noncollinear gradient diffusion directions, with a *b*-value of 1000 s/mm^2^, and one additional volume without diffusion gradients (*b* = 0 s/mm^2^), for the study of brain connectivity.

### MRI analysis: morphometry

3D T1-weighted (T1w) images were analysed both according to an ROI-based and a voxel-based approach, in order to evaluate local and regional volumetric and/or cortical thickness alterations potentially significant for CAS.

In the ROI-based approach, T1w images were segmented in 68 cortical and 19 subcortical structures, by using the FreeSurfer 6.0 recon-all pipeline,^28^ according to the Desikan–Killiany atlas parcellation.^29^ For each ROI, the measures of volume (for subcortical structures) and of cortical morphometry (thickness and volume for cortical areas) were extracted along with the intracranial volume.

In the voxel-wise approach, T1w images were analysed according to the Voxel-Based Morphometry (VBM)^30^ protocol with Diffeomorphic Anatomical Registration using Exponentiated Lie algebra (DARTEL)^31^ algorithm, by using the software package SPM12 (http://www.fil.ion.ucl.ac.uk/spm/software/).

Applying diffeomorphic warping, the DARTEL algorithm allows obtaining a study-specific template from each subject’s brain images and registering these different datasets to this atlas, with an optimal inter-subject realignment also among subjects of different ages. The analysis included the segmentation of brain tissues, the creation of a study-specific template, the calculation of appropriate transformations, the normalization to Montreal Neurological Institute space, and, finally, a standard smoothing using convolution filters (8-mm isotropic Gaussian kernel) to further facilitate matching of volume elements between subjects.

In order to compare the local concentration of grey matter (GM) between CAS and TD, the DARTEL procedure was carried out on the whole sample of 101 subjects (71 CAS + 30 TD), creating an atlas specific for this study.

From tissue segmented maps the mean volumes of global GM, white matter (WM) and cerebrospinal fluid (CSF) were computed. The total intracranial volume (TIV) was calculated as the sum of GM, WM and CSF mean volumes.

### MRI analysis: structural connectivity

Analysis of DWI data was performed in the native space of each subject, by using FSL 6.0.433 and MRtrix software packages.^32^ These software were used to pre-process datasets in order to correct them for head motion, physiological noise artefacts, eddy currents distortions, EPI distortions, and field inhomogeneity. Quality control of head motion was assessed through volume displacement evaluation (FSL 6.0.4)^33^ among DWI volume acquisitions, and a value of 3 standard deviations of the maximum displacement statistics on the entire dataset was imposed as a cut-off for outlier identification. Subsequently, the T1w images were registered to the DWI space using a rigid transformation with FSL's FLIRT command.

Quantitative maps of diffusion invariants such as Fractional Anisotropy (FA) and a colour-encoded image of the main eigenvector were extracted with MRtrix. The FA maps were used to assess the WM integrity of the tracts of interest. On the other hand, the colour-encoded and T1w images helped to identify the seed regions for tract reconstruction in the DWI space. All seed regions were selected manually by a trained researcher (expert in neuroimaging and diffusion studies) and checked by a neuroradiologist and a child neurologist to ensure anatomical accuracy.

Fibre tractography was performed via MRtrix package, by using a probabilistic streamlines method (iFOD2), combined with the constrained spherical deconvolution (CSD) technique to model multiple fibre orientations.^34–36^ In order to correct and increase the anatomical plausibility of the reconstructed fibres, anatomically constrained tractography (ACT) method was applied by using the 5-types-tissue (5TT) images obtained by FreeSurfer segmentation to remove streamlines that were anatomically unfeasible.^37^

Considering the clinical features of CAS in the study of structural connectivity, the following specific tracts of interest were identified and reconstructed in the native space (both in the right and in the left hemisphere):

- The components of the Corticobulbar tract originating from the primary motor cortex in the areas of mouth representation (CBT-Mouth) with a speech-seed ROI in the primary motor cortex just above the Sylvian fissure.^38^ An inclusion ROI was selected in the axial plane between the transverse pontine fibres and the middle cerebellar peduncle.
- The Frontal Aslant Tract (FAT): an oblique bundle in the frontal white matter that connects the inferior frontal gyrus (IFG) with the medial portions of the frontal lobe, namely with the superior frontal gyrus, the cingulate gyrus, the supplementary motor area (SMA) and the pre-supplementary motor area (pre-SMA). For both SMA and pre-SMA components, a seed ROI was placed in the axial plane at the level of the pars opercularis of the inferior frontal gyrus (IFGOp). The SMA inclusion ROI was defined as rostral to the primary motor cortex and caudal to the vertical anterior commissure (VAC) line. The pre-SMA inclusion ROI was defined as rostral to the VAC line and caudal to the virtual line passing through the genu of the corpus callosum.^39^
- The Arcuate Fasciculus (AF): a bundle that is part of the superior longitudinal fasciculus and bidirectionally connects the caudal temporal cortex and the inferior parietal cortex to the frontal lobe and is the main connection between two important speech areas, Broca's and Wernicke's. As in Liégeois *et al*.,^40^ to reconstruct the AF, a seed ROI was selected using three consecutive coronal slices at the level of the arcuate fasciculus bottleneck, an anterior-posterior oriented fibre tract, lateral to the corona radiata and medial to the cortex. The inclusion ROI was delineated in the superior temporal gyrus across three consecutive slices in the axial plane.
- The Inferior Fronto-Occipital Fasciculus (IFOF): a bundle connecting the frontal lobe with the parietal and occipital lobes, passing along the lateral border of the caudate nucleus and the medial part of the corona radiata. To define IFOF, the first ROI was identified in the coronal plane at the middle point between the posterior edge of the cingulum and the posterior edge of the parieto-occipital sulcus. From the coronal slice, the second ROI was delineated for the entire hemisphere at the anterior edge of the genu of the corpus callosum.^41^

For all subjects, the number of streamlines and the mean FA along the tract were calculated for each reconstructed tract (CBT-Mouth, FAT-SMA, FAT-pre-SMA, AF and IFOF). Group analyses were conducted to compare data obtained on subjects with CAS and TD and to identify significant differences between groups in terms of mean FA values along the tracts.

### Statistical analysis

For the ROI-based approach, statistical analysis on the morphometric measures included a two-way analysis of variance (ANOVA), using age, sex and intracranial volume as covariate variables for comparisons between subjects with CAS and TD. A *P*-value of 0.05 was chosen as the significance threshold to reject the null hypothesis of equal means in measurements between groups. A False Discovery Rate (FDR) approach (Benjamini/Hochberg method)^42^ was also applied to take into account the multiple comparisons involved. In the VBM approach, to highlight differences between children with CAS and TD, a General Linear Model (GLM) analysis was performed, in which the concentrations of grey matter were modelled as linear functions of the parameter of interest (group membership of subjects with CAS or TD) and covariate parameters (age, sex and total intracranial volume).

To compare the mean FA values along tracts between the two groups, ANOVA tests (*P* < 0.05) with and without FDR correction were performed using age, sex, tract volume^43^ and number of streamlines as covariates.

Correlation analysis using Spearman’s rank correlation coefficient was then performed in the sample with CAS to explore the relationship between the statistically significant differences in volumetric and connectivity metrics and the speech profile at the group level, taking into account age, sex and manual dominance index as covariate variables.

### Ethics statement

The study was conducted according to the guidelines of the Declaration of Helsinki and was approved by the Regional Pediatric Ethics Committee (CEP) 19-03-2018. The study was conducted in accordance with local legislation and institutional requirements. Written informed consent for participation in this study was provided by the participants’ legal guardians/next of kin.

## Results

### Speech measures

Children with CAS experienced a marked reduction of their phonetic inventory, with a mean number of consonantal speech sounds of 12 out of the 21 (SD = 4), in the Italian repertoire. Their speech production at the single-word level was highly inaccurate (mean phonetic inaccuracy = 62%, SD = 27) and inconsistent (mean phonetic inconsistency = 24%, SD = 18). Syllable omissions accounted for 14% of the productions (SD = 18) and mainly occurred in polysyllabic words. With regards to DDK rate, the children were able to repeat a mean of 21.5 two-syllable sequences (SD = 8.6) and a mean of 12.5 three-syllable sequences (SD = 7.8). Compared to younger TD Italian children, all the speech measures in children with CAS appeared to be markedly below the expected levels for their age.^12^ The mean speech composite severity score was 4.4 (SD = 0.7), thus indicating severe speech alteration.

### MRI structural results and correlations with speech measures

**Figure 1:**
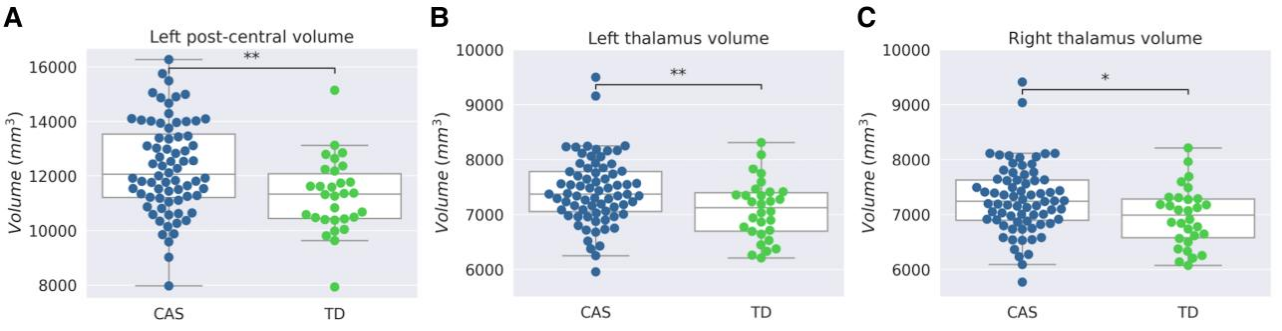
**Cerebral regions of interest with statistically significant increase of volume in children with CAS with respect to TD.** Box plots showing the distributions of volumes corrected for covariates (age, sex and intracranial volume) of the ROIs presenting significant differences between CAS (*N* = 71, blue colour) and TD (*N* = 30, green colour) at ANOVA test with correction for multiple comparisons (FDR). The statistical comparison indicates the significance of the Student’s *t*-test conducted for each ROI between CAS and TD. Panel **A**: left postcentral gyrus volume (*t* = 2.97, *P* = 0.003); panel **B**: left thalamus volume (*t* = 2.67; *P* = 0.008); panel **C**: right thalamus volume (*t* = 2.45; *P* = 0.01). The box ranges from quartile 1 to quartile 3 of the data distribution and the horizontal line drawn inside denotes the median. Regarding the whisker definition, 1.5 times the interquartile range is calculated, and the whiskers are drawn up to the largest observed data point from the dataset that falls within this distance. Each point represents a single subject. * *P* < 0.05; ** *P* < 0.01.

The ANOVA analysis in the ROI-based approach showed significantly greater GM volumes in children with CAS with respect to TD in several ROIs such as the paracentral lobule (left and right), the postcentral cortex (left and right), the precentral cortex (left and right), the pars opercularis (left and right), the precuneus (left and right), the superior parietal cortex (left and right), the superior frontal cortex (left and right), the left temporal pole, the left supramarginal cortex, the left insula, the left posterior cingulate, the right caudal middle-frontal gyrus, the right isthmus of the cingulate and the right lateral orbital cortex. Altered cortical thickness was identified in the postcentral cortex, in the precuneus, in the rostral middle-frontal gyrus, and in the superior parietal cortex. Among the subcortical structures, altered volumes were detected in the left and right thalami. Applying a multiple comparisons correction for FDR, only the increased grey matter volume in the left postcentral volume and in the right and left thalami between children with CAS and TD remains significant at the significance threshold of *P* < 0.05 (Fig. 1).

**Figure 2:**
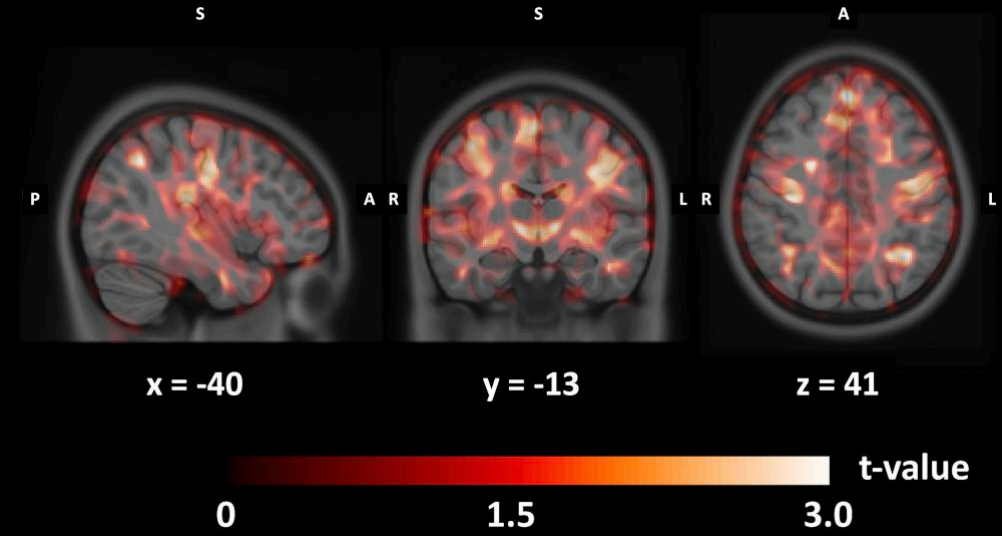
**Results of statistical parametric mapping for the contrast CAS > TD.** Three representative orthogonal slices showing the *t*-statistics map of the comparison between CAS (*N* = 71) and TD (*N* = 30) obtained at the VBM analysis. Red colour scale shows where children with CAS have higher grey matter concentrations than TD.

The VBM analysis showed an increased grey matter concentration bilaterally in the precentral and postcentral gyri, in paracentral lobules, in the superior parietal cortex, and in the posterior cingulate in children with CAS with respect to TD peers. Figure 2 shows the map of *t*-statistics at voxel levels of the comparison between children with CAS and TD. In the Supplementary material, we reported the full description of the statistical parametric mapping analysis, reporting the size of the identified cluster, its statistical significance and its location with reference to the Broadman areas scheme.

**Figure 3:**
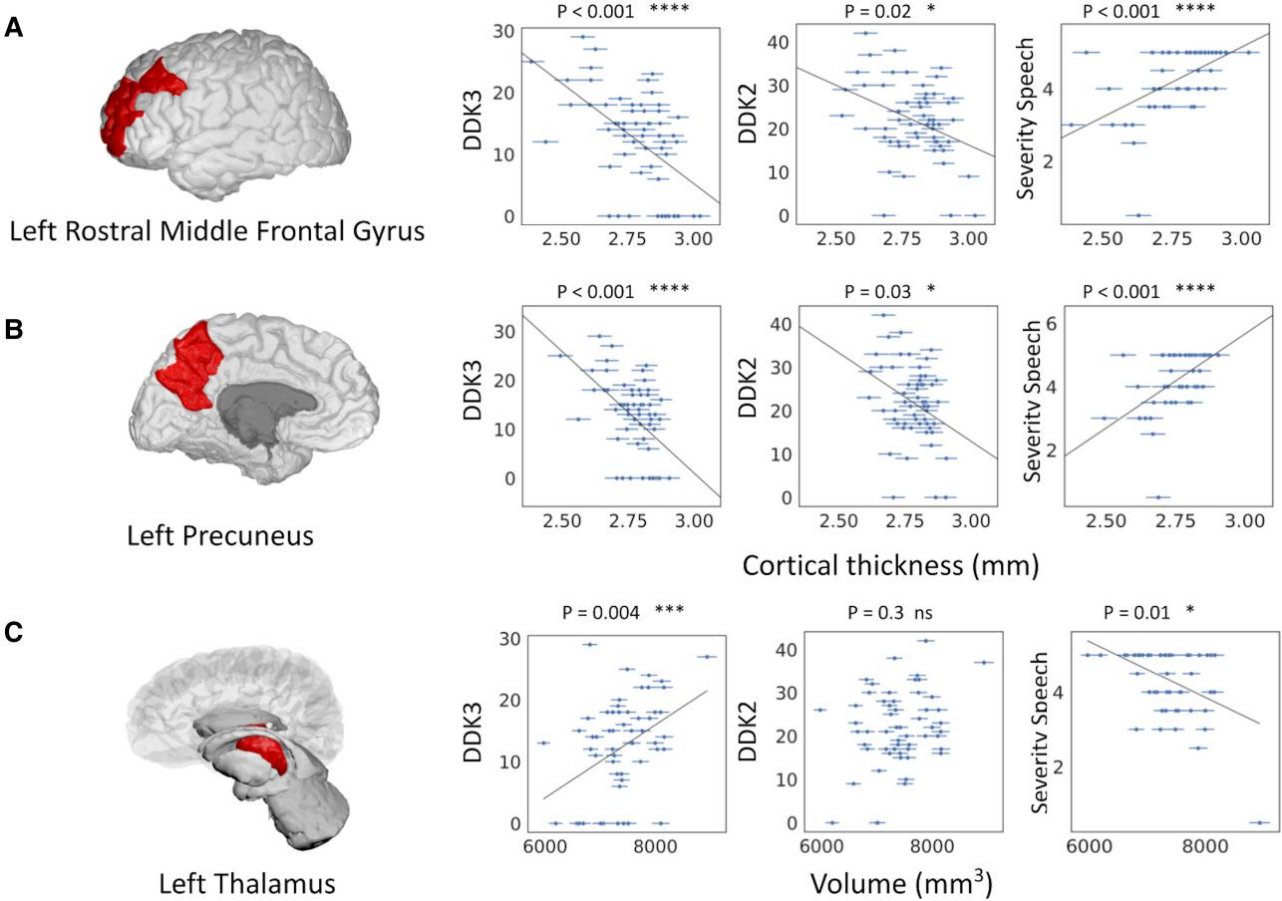
**Correlation analysis between morphometric measures of children with CAS and their clinical speech measures.** Correlation analyses (Spearman’s rank correlation) were conducted in CAS (*N* = 71) between speech measures and morphometric measurements corrected for covariates (age, sex and intracranial volume). Speech measures include DDK3, DDK2 and the speech composite severity score (from left to right). Statistical significance was found for the cortical thickness of the left rostral middle-frontal gyrus (panel **A**; rho = −0.53, *P* < 10–4 for DDK3; rho = −0.31, *P* = 0.02 for DDK2; rho = 0.56, *P* < 10–5 for speech composite severity score), the cortical thickness of the left precuneus (panel **B**; rho = −0.51, *P* < 10–4 for DDK3; rho = −0.29, *P* = 0.03 for DDK2; rho = 0.55, *P* < 10–4 for speech composite severity score), and the volume of the left thalamus (panel **C**; rho = 0.37, *P* = 0.004 for DDK3; rho = 0.14, *P* = 0.3 for DDK2; rho = −0.33, *P* = 0.01 for speech composite severity score third row). Each point represents a single subject. The error bars for MRI measurements correspond to the estimated error reported in literature by the authors of the segmentation method, for cortical regions^44^ and for the thalamus in young adults.^45^ * *P* < 0.05; ** *P* < 0.01; *** *P* < 0.005; **** *P* < 0.001; ns = not significant.

To study possible brain structure-function relationships, we analysed, in CAS, the correlations of some speech measures (DDK3 and DDK2) and of the speech composite severity score with cortical thickness and volumetric values, which were found to differ significantly from the TD control group, including those that did not survive after FDR correction. Cortical thickness measures for the left rostral middle-frontal gyrus and the left precuneus, as well as the volume of the left thalamus, correlated with clinical data (Fig. 3). Namely, the cortical thickness of the left middle-frontal gyrus was found to be significantly and negatively correlated with DDK3 (*P* < 0.001) and DDK2 (*P* = 0.02), and it also correlated positively with the speech composite severity score (*P* < 0.001). Similar results were also found for the cortical thickness of the left precuneus, which correlated negatively with DDK3 (*P* < 0.001) and DDK2 (*P* = 0.03) and positively with the speech composite severity score (*P* < 0.001). On the other hand, with regard to the volume of the left thalamus, opposite results were found and namely, it correlated positively with DDK3 (*P* = 0.004) and negatively with the speech composite severity score (*P* = 0.01), suggesting that, in this case, better performance is associated with greater volume of the thalamus.

### Structural connectivity results and correlations with speech measures

**Figure 4:**
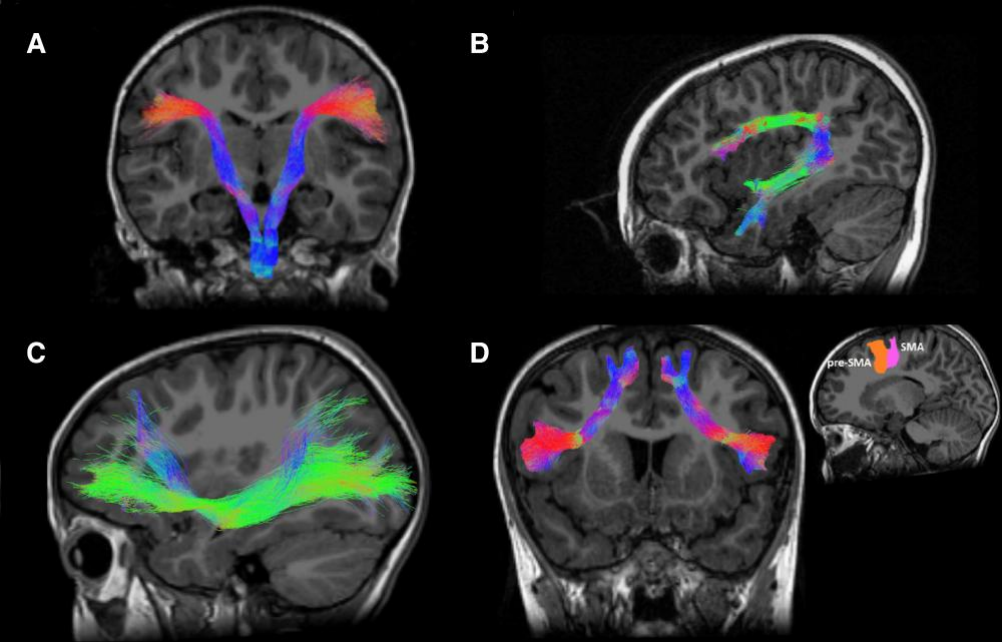
**Representation of the bundles of interest, selected for a participant with CAS. (A**) The Corticobulbar Tract originating from the motor cortex of the mouth (CBT-Mouth) (**B**); the Arcuate Fasciculus; (**C**) the Inferior Fronto-Occipital Fasciculus; (**D**) the Frontal Aslant Tract, with the two components pointing to the supplementary motor area (SMA) and the pre-supplementary motor area (pre-SMA). These two areas are represented in the sagittal image with the SMA in pink and the pre-SMA in orange. The colour code of the tracts corresponds to the standard RGB code, where each colour represents the direction of water diffusion (red for Right-Left direction, green for Anterior-Posterior, and blue for Superior–Inferior).

At data pre-processing stage, two DWI datasets from children with CAS were discarded for excessive movement, while other two datasets were excluded during post-processing, so the final sample for structural connectivity analyses consisted of 67 subjects with CAS (53 males, 14 females, mean age = 6.0 ± 2.1 years; range = 2.8–11.2 years) and 30 TD subjects (18 males, 12 females, mean age = 6.5 ± 2.6 years; range = 2.6–12.7 years). For each participant in these two groups, the fibre tracts of interest were extracted. Figure 4 presents the five reconstructed tracts of one representative case.

**Figure 5:**
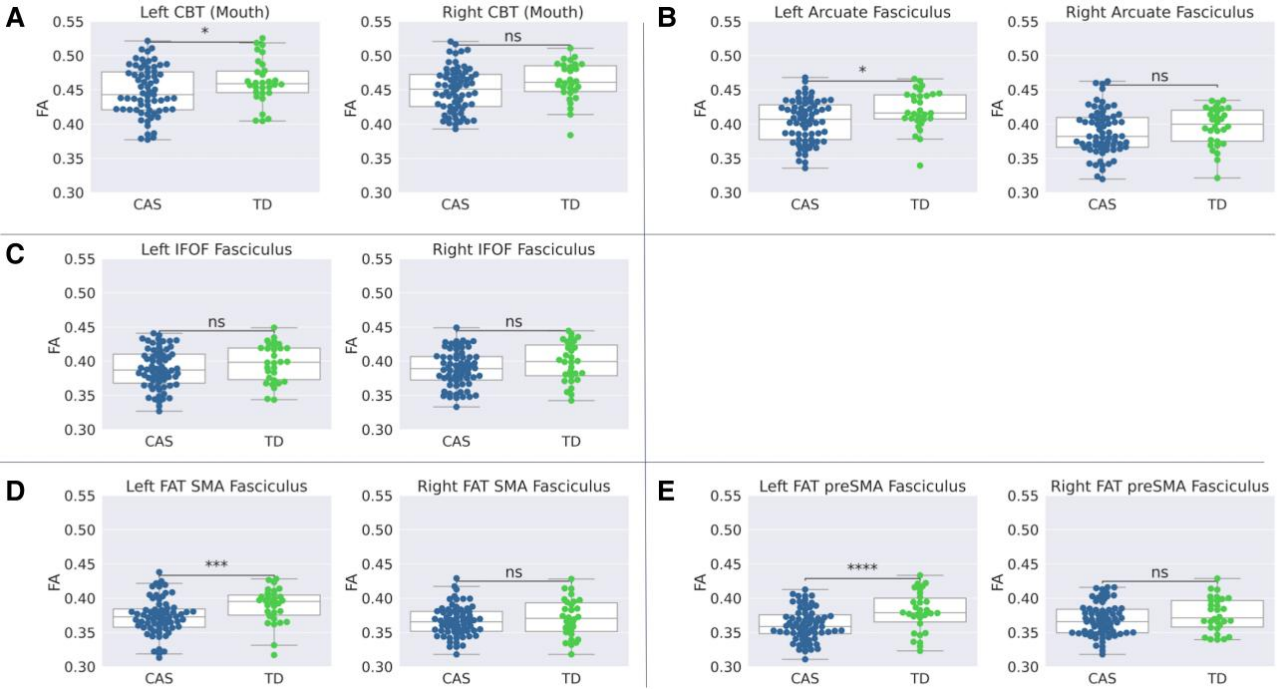
**Statistical comparison of fractional anisotropy distributions between CAS and TD along the tracts of interest.** Boxplot of the mean FA values calculated along the tracts of interest for CAS (*N* = 67, blue colour) and TD (*N* = 30, green colour) groups. The asterisks indicate the significant differences obtained by the ANOVA analysis without FDR. Panel **A**: Corticobulbar Tract-Mouth (*P* = 0.02 for left and *P* = 0.05 for right); panel **B**: Arcuate Fasciculus (*P* = 0.01 for left and *P* = 0.19 for right); panel **C**: IFOF (*P* = 0.12 for left and *P* = 0.07 for right); panel **D**: FAT-SMA (*P* = 0.001 for left and *P* = 0.33 for right); panel **E**: FAT-pre-SMA (*P* = 0.0001 for left and *P* = 0.10 for the right). The description of the elements of the box plots is reported in the caption of Fig. 1. Each point represents a single subject. IFOF, Inferior Frontal Occipital Fasciculus; FAT, Frontal Aslant Tract; SMA, Supplementary Motor Area. * *P* < 0.05; ** *P* < 0.01; *** *P* < 0.005; **** *P* < 0.001; ns = not significant.

The comparison between the CAS and the TD group, via the ANOVA analysis, showed statistically significant differences of FA values along the left components of the CBT-Mouth (*P* = 0.02), the arcuate fasciculus (*P* = 0.01), and both FAT bundles (*P* = 0.001 and *P* = 0.0001, for tracts pointing to SMA and pre-SMA, respectively) (Fig. 5). In all these tracts, children with CAS showed lower FA values than TD peers. No significant differences in FA were found in all the right hemisphere bundles, nor in the IFOF tract of both hemispheres.

Applying FDR correction, only the differences of FA in both left FAT bundles (SMA and pre-SMA) survived.

**Figure 6:**
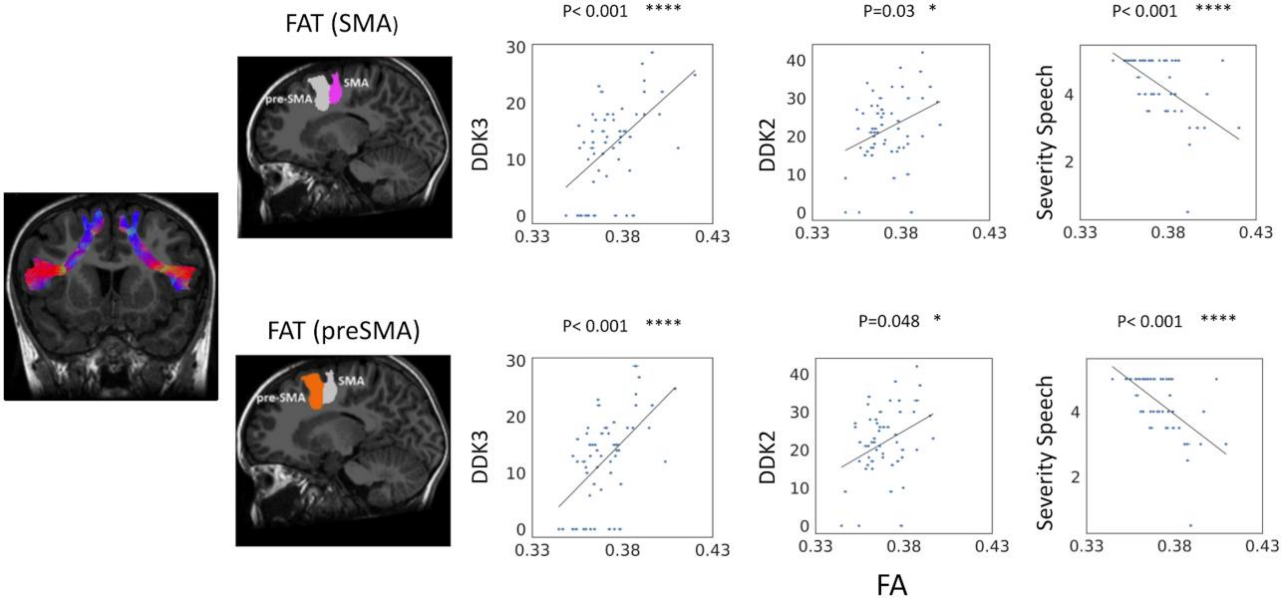
**Correlation analysis between mean FA values along tracts in children with CAS and the clinical speech measures.** Results of correlation analysis (Spearman’s rank correlation) between significantly different FA values along the two components of left FAT and the clinical parameters of DDK3, DDK2 and the speech composite severity score (from left to right) of each subject with CAS (*N* = 67). Top row: left FAT-SMA (rho = 0.53, *P* < 10–4 for DDK3; rho = 0.29, *P* = 0.03 for DDK2; rho = −0.56, *P* < 10–5 for speech composite severity score first row); bottom row: left FAT-pre-SMA (rho = 0.54, *P* < 10–4 for DDK3; rho = 0.27, *P* = 0.048 for DDK2; rho = −0.60, *P* < 10–6 for speech composite severity score first row). Each point represents a single subject. The error bars for FA correspond to the standard error calculated as the ratio of the standard deviations of FA values on the reconstructed tracts and the square root of the number of voxels of the reconstructed fasciculus. * *P* < 0.05; ** *P* < 0.01; *** *P* < 0.005; **** *P* < 0.001.

For these two bundles, we studied, in CAS, the correlations of the mean FA values with the speech measures. The results are shown in Fig. 6. It can be observed that FA values of both components of the left FAT show a significant positive correlation with the DDK3 (*P* < 0.001 and *P* < 0.001 for tracts pointing to SMA and pre-SMA, respectively). DDK2 correlates positively with the FA in the FAT component, pointing to SMA (*P* = 0.036) and to pre-SMA (*P* = 0.048). Finally, the speech composite severity score correlates significantly and negatively with the FA of both components of the left FAT (*P* < 0.001 and *P* < 0.001 for SMA and pre-SMA, respectively).

## Discussion

Morphometric brain imaging investigation and deep speech phenotyping were carried out on a large sample of 71 Italian children with CAS to detect volumetric alterations and their possible associations with impaired speech performances. DWI datasets from 67 of these subjects allowed the study of structural connectivity, with a particular focus on five tracts of interest: the corticobulbar mouth tract, the arcuate fasciculus, the inferior fronto-occipital fasciculus and the two components of the frontal aslant tract. Correlations between speech performances and statistically significant volumetric and connectivity measures were investigated. Concerning the morphometric analysis, uncorrected results revealed a widespread alteration in children with CAS in terms of an increase in cortical thickness and GM volume in several cortical and subcortical structures. As hypothesized, some of these alterations involved areas which are crucial for sensorimotor speech processing and were already documented in children with CAS: for instance, those involving the precentral cortex bilaterally,^46^ and the left supramarginal cortex.^13,14,22^ Interestingly, the increase of grey matter in the postcentral gyrus and in the thalami (left and right) remained significant, also setting a more stringent significance threshold at *P* < 0.05 corrected for multiple comparisons. These results might suggest that in our children with CAS, the altered maturational pattern of the CNS mainly affects areas and structures that are relevant for sensorimotor control, rather than those involved in higher-order associative functions. The postcentral gyrus, as parcellated according to the Desikan-Killiany atlas used in this study, contains the primary somatosensory cortex, in which tactile and proprioceptive stimuli are received, including those from the face, lips and tongue. During the early stages of typical speech development, the somatosensory feedback has been hypothesized to contribute to the establishment of robust neural programmes that encode the sensory consequences of the articulators’ movements.^47^ According to the Directions Into Velocities of Articulators (DIVA) neurocomputational model of speech motor learning and control, babbling consists of randomly generated speech-like movements and sounds. During early speech development, auditory and somatosensory feedback from babbling practice is used to map speech sounds in the premotor cortex, thus enabling the feedforward control mechanism, whilst information collected in the error maps of the auditory and somatosensory cortices supports the refinement of speech motor output via a feedback mechanism.^7,47,48^ Based on the DIVA neurocomputational model, altered somatosensory processing may thus impact speech acquisition. In line with this model, results from the current study reveal altered grey matter in the postcentral gyrus and thalamus, which have a fundamental role in somatosensory processing. Though the sensory profiles of children with CAS and, in particular, their tactile and proprioceptive sensitivity, represent a scarcely investigated topic, it is hypothesized that children with CAS might endure reduced oral somatosensory sensation of the tongue and palate,^5^ possibly as part of a more widespread sensory integration dysfunction with hypo- or hypersensitivity.^1,49–51^ These alterations might play a role in decreasing the somatosensory feedback during speech production and hinder the sound-to-body construction of a multimodal, integrated speech sound map, starting from the babbling phase, that is conceived as the foundation for speech and language development. In our previous study, canonical babbling was found to be absent or abnormal in up to 85% of the children with CAS^11^ and sporadic and scarcely varied babbling is commonly reported as an important early sign of increased risk of CAS,^52–55^ that may reflect a disrupted mapping between auditory, somatosensory and motor experiences. Moreover, sensory alterations would possibly play a role in preventing the shift to efficient feedforward control strategies and force children with CAS to rely on slow, resource-consuming, pre-emptive and reparatory control strategies, primarily based on auditory feedback.^56^ The postcentral gyrus also houses the secondary somatosensory cortex, which appears to play a role in the integration of somatosensory stimuli, memory formation and learning.^57^ In particular, the secondary somatosensory cortex is active when attention is paid to a tactile stimulus in order to locate it and to gauge intensity for further higher-order elaborations.^58,59^

As for the analysis of subcortical structures, we found a significantly increased volume of the thalamus bilaterally in children with CAS. The thalamus is a complex structure, richly connected to the sensory, as well as to the prefrontal motor cortices, whose role in speech generation and motor control is well known. It is also included in the DIVA model as part of the feedback mechanism for speech motor control.^7^ The thalamus appears to play a role in informing several cortical areas of an upcoming motor command and in providing an anticipatory model of the expected sensory consequences of a motor response.^60^ Beyond the suggested implications of the thalamus in motor control and its role as a relay station for multimodal sensory inputs, some authors also highlight its functional contributions to distinct higher-order cognitive and executive skills, such as working memory, new learning, adaptive decision making, attention and several language functions across phonology, lexical-semantics, morphology and syntax.^61–63^

With regard to structural connectivity, as hypothesized, the analysis revealed statistically significant differences between children with CAS and controls in pathways already found to be altered in CAS. However, only differences in FA along both components of the FAT survived after correction for multiple comparisons. The FAT plays an important role in planning, timing, and coordination by resolving competition between sequential motor plans.^25,27^ In particular, damage to the left FAT was associated with apraxia of speech in aphasic adults with left hemisphere ischaemic lesions,^64^ thus lending support to the hypothesis that, in this case, speech motor skills rely on the integrity of white matter via the FAT, rather than on that of the classical speech–language areas. Indeed, our present findings confirm in a larger sample of children with CAS the role of altered connectivity of the left FAT, not only in the pre-SMA, as found in Bombonato *et al*.,^24^ but also in the SMA component. The FAT has been primarily studied in adults undergoing surgery for brain tumours,^26,65–67^ with post-stroke aphasia,^68^ or with primary progressive aphasia,^69,70^ and less frequently in children without brain lesions. However, there is evidence that in the paediatric population, FAT dysfunction is related to developmental stuttering.^27,71,72^ These results lend support to the importance of FAT integrity in speech fluency, rate, and initiation. As a matter of fact, this white matter bundle connects the IFG to the SMA/pre-SMA, both areas being crucial for speech. The SMA and pre-SMA have been associated with high-order selection and execution in both speech and nonspeech domains,^73–75^ and a lesion in these areas can lead to motor and speech deficits.^76,77^ The IFG comprises the classical Broca’s area, in particular the pars opercularis and triangularis, whose role in speech, as well as in language production, is well known. Our results of FAT connectivity alterations in CAS highlight the importance, also in the developing brain, of this tract integrity, as one of the neural underpinnings of the core deficit involving speech motor planning and programming.

### Correlations with speech measures

Investigating the relationship between morphometric and speech measures and overall severity, the significantly increased cortical thickness of the left rostral middle-frontal gyrus and left precuneus, as well as the increased volume of the left thalamus, correlated with some speech measures and with the overall severity of CAS. Increased cortical thickness of cortical areas was associated with worse speech performances, in particular with poorer DDK rate and with higher speech severity composite score. The precuneus appears to be involved in planning during lexical selection^78^ and in the initiation of action,^79^ and is generally considered to be directly involved in functions that require a complex level of integration.^80^ The rostral middle-frontal cortices have been hypothesized as a site of convergence of the dorsal and ventral attention networks^81^ and are involved in word retrieval and in managing working memory. Conversely, the increased volume of the left thalamus is associated with better speech performance. Pigdon *et al*.^82^ in an fMRI study described increased activation at the level of subcortical structures, namely the thalamus, in children with SSD (not CAS) during a non-word repetition task. Children with SSD, differently from typical controls, showed a greater role of subcortical structures, which was interpreted by the authors as a possible compensation for the altered functioning of the classical speech/language lateral cortices.

With regards to the correlations between FAT connectivity and speech measures, better speech performances were associated with higher FA in both components of the left FAT (SMA and pre-SMA). Indeed, we found a significant positive correlation with the DDK2 and DDK3: higher FA along the left FAT is associated with a higher number of repetitions of the non-significant strings, indicating better speech motor sequencing. Conversely, decreased connectivity in the left FAT is related to worse overall speech severity. These findings confirm a brain-function correlation in children with CAS and may represent an important step forward in the understanding of the neural basis of this persistent and impactful disorder.

### Limitations and future directions

One limitation is that TD children did not undergo formal speech, language and cognitive assessment. Their typical speech, language and academic achievements were inferred from reports by parents and school teachers. Given that our results suggest the involvement of the thalamus in CAS, it would be interesting to study in detail this subcortical structure: at structural level, locating the thalamic subnuclei more altered in CAS respect to TD, and investigating their connections with cortical areas, and at functional level, exploring the functional connectivity of these structures in children with CAS. Finally, it would be interesting to study in-depth the somatosensory profiles of children with CAS, along with their speech and language and cognitive functioning, as a better understanding of their sensory characteristics could bring important implications for treatment, overall management in educational settings, and to foster their autonomy and well-being. A possible future research perspective could be to study the association between the early signs of disrupted speech acquisition, such as the scarcity or lack of canonical babbling, and the neural CAS biomarkers, in order to allow early intervention and support at the age of maximum neural plasticity.

## Conclusion

Our results provide neural evidence of sensorimotor deficits in a large sample of children with CAS compared to TD peers. Indeed, morphometric analysis revealed alterations in areas and structures involved in somatosensory processing, whilst the connectivity study highlighted the role of the FAT as a white matter bundle whose integrity is salient for motor speech control. After correction for multiple comparisons, the volumetric alterations specifically involved the left primary sensory cortex and the thalamus bilaterally. These findings are consistent with reports on the presence of widespread sensory alterations in children with CAS, especially with regard to intraoral tactile sensation. Sensory dysfunction could be present early on, starting from the babbling phase and possibly could explain weak speech motor maps with difficult integration of auditory and somatosensory information due to developmental neuroanatomical atypicalities. Increased grey matter volumes in children with neurodevelopmental disorders are typically interpreted either as a sign of delayed maturation of the CNS or as the result of compensatory neuroplastic mechanisms. Disentangling which of the two effects applies to children with CAS is challenging; however, we believe that our findings could potentially have implications for clinical management of CAS. Abnormalities in structures underlying sensorimotor processing and somatosensation point to the importance of using multimodal treatment approaches that include consistent somatosensory inputs and may further explain why children with CAS need intensive speech motor practice to build up and control functional synergies for speech production. Moreover, in our study, children with CAS showed lower connectivity in the left FAT, pointing at the SMA and pre-SMA. The study of the FAT is promising to understand the neural basis of motor speech disorders, and its relevance for speech planning and programming is supported by the correlation between the severity of the speech disorder and its connectivity in our sample. The results of the present study are consistent with data from the literature and further confirm that the integrity of this tract is crucial for fluency, rate, and language initiation, as well as for broader cognitive functions.

## Supplementary Material

fcaf302_Supplementary_Data

## Data Availability

Data and all the materials are available in anonymous form upon reasonable request by contacting the corresponding author (L.B.) at the following link: https://doi.org/10.5281/zenodo.15638618.
