# Supplementary material for: Alterations of the neural substrate in childhood apraxia of speech: new evidence from neuroimaging
Source: Brain Commun. 2025 Sep 16;7(5):fcaf302. doi: 10.1093/braincomms/fcaf302 (PMC12448706; doi:10.1093/braincomms/fcaf302)
Supplement: fcaf302_Supplementary_Data [file fcaf302_supplementary_data.pdf]

Supplementary Table 1. Grey matter areas showing statistically significant differences between children with CAS and TD (CAS > TD); thresholds: p (un-corrected) < 0.005; cluster size >5

Area Name	Brodmann Area (BA)		Number of Voxels	MNI Coordinates			Significance		
				x	y	z	Z score	t - value	p-value (uncorr)
Middle Frontal Gyrus - Inferior Frontal Gyrus	44-9	RH	180	29	12	29	3.69	3.84	<0.001
PreCentral Gyrus - Middle Frontal Gyrus	6	LH	38	-36	-10	38	2.93	3.01	0.002
PostCentral Gyrus - PreCentral Gyrus	4-3	RH	186	38	-21	44	3.39	3.51	<0.001
		LH	6	-45	-20	44	2.72	2.78	0.003
Cingulate Gyrus -Medial Frontal Gyrus	24-32	RH	183	20	4	48	3.88	4.04	<0.001
Medial Frontal Gyrus	6	IH	338	0	-22	64	3.54	3.66	<0.001
	6	IH	50	0	4	56	3.19	3.29	0.001
	8-9	IH	109	0	45	40	3.41	3.52	<0.001
	9-10	IH	21	0	57	24	2.97	3.04	0.002
Anterior Cingulate Gyrus -Medial Frontal Gyrus	10	IH	25	0	62	6	3.21	3.30	0.001
		RH	76	12	51	-9	2.96	3.04	0.002
PreCuneus	7	RH	161	8	-64	36	3.34	3.45	<0.001
		LH	11	-15	-50	50	2.78	2.85	0.003
Superior Parietal Lobule	7	RH	39	27	-54	56	3.08	3.16	0.001
Inferior Parietal Lobule	40	LH	91	-39	-57	42	3.16	3.26	0.001
		RH	10	39	-54	45	2.75	2.81	0.003
Insula - Superior Temporal Gyrus	40-41	LH	5	-40	-27	18	2.64	2.70	0.004
Thalamus		LH	108	-14	-22	-3	3.32	3.42	<0.001
		LH	14	-15	-6	-6	2.69	2.75	0.004
		RH	40	20	-6	-4	2.88	2.95	0.002
		RH	21	10	-12	-2	2.82	2.88	0.002

LH = Left Hemisphere; RH = Right Hemisphere; IH = Inter-Hemispheres
